# Serological Screening and Risk Factors Associated with *Leishmania infantum* Positivity in Newly Diagnosed HIV Patients in Greece

**DOI:** 10.3390/microorganisms12071397

**Published:** 2024-07-10

**Authors:** Chrysa Voyiatzaki, Apollon Dareios Zare Chormizi, Maria E. Tsoumani, Antonia Efstathiou, Konstantinos Konstantinidis, Georgios Chrysos, Aikaterini Argyraki, Vasileios Papastamopoulos, Effie G. Papageorgiou, Marika Kotsianopoulou

**Affiliations:** 1 Department of Biomedical Sciences, Division of Medical Laboratories Science, University of West Attica, 12243 Athens, Greece; 2 Immunology of Infection Group, Department of Microbiology, Hellenic Pasteur Institute, 11521 Athens, Greece; 3 Laboratory of Biology, Department of Medicine, Democritus University of Thrace, Dragana, 68100 Alexandroupolis, Greece; 4 Second Department of Internal Medicine, Tzaneio General Hospital of Piraeus, 18536 Athens, Greece; 5 Department of Internal Medicine, Sotiria Thoracic Diseases General Hospital, 11527 Athens, Greece; 6 Infectious Diseases Unit, 5th Department of Internal Medicine, Evaggelismos General Hospital, 10676 Athens, Greece; 7 Department of Public Health Policy, School of Public Health, University of West Attica, 11521 Athens, Greece

**Keywords:** co-infection, Europe/Greece, HIV, intravenous drug users, leishmaniasis, seroprevalence, tropical neglected disease

## Abstract

A serological screening was conducted to detect IgG antibodies against *Leishmania infantum* (*L. infantum*) in newly diagnosed human immunodeficiency virus (HIV) patients in Greece. The study also examined potential risk factors and the agreement of commercially available serological methods. IgG antibodies against *L. infantum* were detected using enzyme-linked immunosorbent assay (ELISA), indirect immunofluorescence antibody test (IFAT), and Western blot (WB). Out of 155 samples, 14 (9.0%) tested positive for IgG antibodies against *L. infantum* using at least two methods. Statistical analysis showed substantial agreement between WB and IFAT methods (Cohen’s kappa = 0.75) but moderate overall agreement among the three methods (Fleiss’ kappa = 0.42). Additionally, HIV+ intravenous drug users faced 3.55 times (*p* = 0.025) higher risk of testing positive for *L. infantum* IgG, positing that anthroponotic transmission between these patients is a plausible hypothesis based on existing literature. Non-invasive and cost-effective techniques are preferred to detect asymptomatic infections, and leishmaniasis screening should be conducted immediately after HIV diagnosis in endemic regions to enable prophylactic treatment for leishmaniasis in addition to antiretroviral therapy. To maximize sensitivity, performing at least two different serological methods for each patient is recommended.

## 1. Introduction

It is estimated that between 30.2 and 45.1 million individuals across the globe were living with human immunodeficiency virus (HIV) in 2020, and there were 1.5 million new cases reported in that same year [1]. In Greece, 601 individuals were diagnosed with HIV in 2020 [2], and 526 new cases were reported in 2021, bringing the total number of HIV diagnoses in Greece to 19,265 [3]. Leishmaniasis is a common parasitic infection and disease in Europe, and co-infection with HIV can result in intricate interactions among pathogens that are not fully comprehended. However, their interactions have been shown to accelerate the development of conditions that define acquired immune deficiency syndrome (AIDS), trigger latent parasitic infections, and induce uncontrolled proliferation of parasites or facilitate the establishment of a primary parasitic infection due to the pre-existing immunosuppression caused by the virus. Based on existing published literature, the incidence of *Leishmania*–HIV co-infection is considered to be infrequent in Greece, with only a few documented cases [4,5].

Leishmaniasis is a neglected parasitic disease that is commonly found in tropical regions and is caused by a minimum of 20 distinct species of the genus *Leishmania*. Over 90 species of sand fly vectors transmit the disease to around 70 mammalian hosts, including humans [6,7]. In the Mediterranean region, the species *Leishmania infantum*, *Leishmania donovani*, *Leishmania major*, and *Leishmania tropica* are found and are transmitted by sand flies of the genus *Phlebotomus* [6]. *L. infantum* is the most common causative agent of visceral leishmaniasis (VL) in this region [8], and dogs are the primary reservoir, with a median seropositivity of 10% [9]. The disease typically presents with symptoms such as fever, hepatosplenomegaly, and pancytopenia [10].

There are an estimated 50,000 to 90,000 new cases of VL worldwide each year [7]. Incidence numbers are most likely underestimated due to no mandatory reporting in many endemic regions [11]. The disease is endemic in the Mediterranean region, accounting for 5 to 6% of the global burden, with an estimated 1200 to 2000 new cases yearly [12]. In Southern Europe, seroprevalences ranging between 10 and 47% have been found, and it is believed that many people have asymptomatic infections with the protozoan *L. infantum*. The first asymptomatic infection was documented in Italy between 1971 and 1972 [13,14]. The challenge of diagnosing and treating the disease is increased because many infections are sub-clinical, making it difficult to control the disease in endemic regions [15].

Over the past decade, there has been a significant decrease in reported cases of VL, partly attributed to the natural cycles of the disease. Currently, the disease is mainly found in Albania, Georgia, Italy, and Spain within the European region [16]. Nonetheless, there is an urgent need for clinical vigilance and surveillance as leishmaniasis spreads to northern regions and outbreaks re-emerge in endemic areas. Additionally, there is a risk of introducing new strains or species of *Leishmania* due to the movement of populations [12,13].

VL–HIV co-infection presents an important challenge for VL control and is a severe threat to public health. In regions where VL is endemic, individuals co-infected with HIV are 100 to 2320 times more likely to develop VL. Furthermore, between 2 and 12% of cases of VL have been reported in HIV+ patients [17,18].

In Greece, leishmaniasis is a notifiable disease that has been under the surveillance of the Greek Ministry of Health since 1961 [19]. In 1998, it was incorporated into the mandatory declaration system of the Greek National Public Health Organization (NPHO) [16]. Despite being endemic in the country, occurrences of VL–HIV co-infection are rare, with only a minimal number of cases being documented [4,5].

VL–HIV co-infection was initially reported in 1985, and since then, the number of cases in Southern Europe has increased rapidly [17]. However, the use of highly active antiretroviral therapy (HAART) has led to a progressive decrease in primary VL infections in HIV+ patients since 2001 [20]. Although asymptomatic infection has been found in many patients receiving HAART, this does not appear to improve the clinical outcome of the disease [12,21,22]. As of 2021, despite reports of co-infection in 45 countries [7], there is still no provision for leishmaniasis screening in HIV+ patients who live in or visit endemic areas [6].

VL–HIV co-infections have been predominantly reported in France, Italy, Portugal, and Spain, primarily in densely populated coastal urban areas where intravenous drug use has become a significant social issue. Spain has reported the highest number of VL cases, with approximately 70% occurring among intravenous drug users [10]. In Europe, a possible direct transmission cycle through the sharing of syringes among HIV+ intravenous drug users has been reported [6,10].

Patients with VL–HIV co-infection exhibit atypical VL clinical signs and the absence of splenomegaly, which makes leishmaniasis challenging to diagnose. They also have high parasite levels, increased relapses and reinfections, low cure rates, and high mortality rates compared to those without HIV [17,20,21,22]. Despite successful treatment for leishmaniasis, the parasite persists in the HIV+ or HIV− patient for life [22,23]. Patients with co-infections have a higher expression of inhibitory molecules in CD4+ T-cell surfaces, contributing to a higher parasite burden even after successful treatment [20].

Around 80% of VL–HIV co-infected patients experience at least one relapse, which is higher than the general population [24]. Predictors of relapse include a history of relapse, not receiving secondary prophylaxis, and not recovering normal CD4+ cell levels [25]. Specifically, a CD4+ cell count below 200/μL serves as a strong predictor of relapse and development of clinical disease, regardless of receiving HAART or secondary prophylaxis [12,26]. Even patients with high CD4+ cell counts under HAART with immune reconstitution and undetectable HIV RNA levels have reported relapses [17,27].

In the case of VL–HIV co-infection, both pathogens establish a symbiotic relationship by interacting through a common immunopathological mechanism involving dendritic cells and macrophages. However, the precise mechanisms of interaction remain unclear [26,28]. It has been observed that HIV can activate latent VL infection or facilitate primary infection, leading to a higher risk of VL progression in HIV+ patients [18,22,24]. Additionally, VL infection can activate the latent phase of HIV [17], causing its multiplication and leading to the development of AIDS-defining conditions [17,28,29]. VL has been identified as an opportunistic infection in HIV+ patients and is categorized as an AIDS-defining stage 4 disease in the World Health Organization (WHO) HIV/AIDS classification [6].

Regarding laboratory diagnosis, the efficacy of laboratory methods for diagnosing VL in HIV patients has been the subject of limited available data, leading to inconsistent results, as reported in various studies [20,30,31].

The gold standard for diagnosing VL is the detection of amastigotes in a bone marrow or spleen biopsy through microscopic examination using Giemsa and hematoxylin and eosin stains [15,17]. However, this method is invasive and may pose life-threatening complications [32]. Furthermore, the biopsy method may not be sensitive enough to identify all cases of VL [15,33], leading to false-negative results due to several factors, such as low *Leishmania*-infected cells due to pancytopenia or previous treatment with pentamidine or amphotericin B [17], or parasites located in atypical sites [32,34]. Therefore, less invasive diagnostic methods should be preferred, especially in cases of relapse, a phenomenon commonly observed in patients with VL-HIV co-infection. Molecular methods are efficacious in the diagnosis of VL and in monitoring the treatment outcomes of infected patients [17]. However, it is uncertain whether they can accurately detect asymptomatic infections in co-infected patients [33]. A study reported the detection of *Leishmania* spp. antibodies in 26 out of 163 HIV+ patients. Of these, only three patients had tested positive for kDNA-polymerase chain reaction (PCR) in their peripheral blood samples [35]. Furthermore, their usage in regions with a high prevalence of co-infections is limited due to the expensive nature of these tests and their requirement for advanced laboratory settings [15,33].

When definitive diagnostic tests for VL (detection of amastigotes by microscopy) cannot be conducted or have negative results, serologic testing is a recommended alternative for patients [15]. Serological tests are simple, non-invasive techniques. Several immunological serological methods, such as enzyme-linked immunosorbent assay (ELISA), indirect immunofluorescence antibody test (IFAT), and Western blot (WB), can be utilized to detect IgG antibodies against *L. infantum*. A systematic review and meta-analysis of these methods’ overall sensitivities and specificities in patients with *Leishmania*–HIV co-infection noted that the overall sensitivity was low, while the overall specificity was high [33]. The study did not include asymptomatic patients, and as the authors note, its validity relies on scarce evidence that may change as more extensive, well-designed studies are conducted. The calculated sensitivities and specificities of these methods in patients with *Leishmania*–HIV co-infection were as follows: ELISA—66% sensitivity and 90% specificity, IFAT—51% sensitivity and 93% specificity, and WB—84% sensitivity and 82% specificity [33].

The ELISA assay has demonstrated sensitivity in detecting *Leishmania* infection, yet its specificity varies depending on the type of antigens employed [30]. Recombinant antigens are considered more accurate than soluble ones [15]. The most reliable recombinant antigen is rK39 (recombinant 39 amino acid kinesin antigen) [23]. ELISA rK39 positivity is typically associated with active disease [36]. Tests that utilize crude antigens may result in false positives due to cross-reactivity with mycobacteria [15,32]. False negatives can occur in the early stages of infection or asymptomatic patients. Detectable antibody levels do not indicate parasite load [30].

VL cases in South America are primarily concentrated in Brazil, accounting for 96% of the total cases. The Brazilian VL surveillance and control program has been using the IFAT test for diagnosing VL for the past few decades [31]. The IFAT test employs antigens from axenic cultures of promastigotes or smears of organs with amastigote forms [32]. However, this test has some limitations, including cross-reactivity with trypanosomatids and its failure to identify asymptomatic infections [30]. Nevertheless, in Brazil, the possible cross-reactivity with trypanosomatids (3.1%) does not appear to mislead the overall prevalence of co-infection [35]. The diagnostic accuracy of the IFAT method using *L. infantum* promastigotes has been observed to be high in patients with *L. infantum*–HIV co-infection, particularly with the 1/80 dilution [15]. A systematic review and meta-analysis estimated IFAT to have low sensitivity and specificity, but only 6 out of 33 IFATs used in the included studies employed *L. infantum* antigens [33].

The WB technique is a highly sensitive (76–90%) and specific (94–100%) test superior to IFAT and ELISA. It can confirm a positive result obtained from another serological test and even identify false-positive results obtained by ELISA [30,32,33]. Combining it with other serological methods is valuable in diagnosing VL in patients with HIV co-infection [34]. However, a weak zonal signal is often observed due to the reduced immune response of HIV+ patients [10]. The 16 kDa antigenic component is the most sensitive and specific diagnostic zone [33], and the presence of antibodies reacting with *L. infantum* antigens of 14 and/or 16–18 kDa not only indicates acute VL but may also be evidence of delayed-type antileishmanial hypersensitivity in asymptomatic individuals [32].

If the diagnosis is to be serological, to maximize sensitivity it is recommended to perform at least two different methods for each patient, such as IFAT and WB [17,32].

In this study, a serological screening was conducted to identify IgG antibodies against *L. infantum* in newly diagnosed HIV+ patients under surveillance in Greek infectious disease units who had not received treatment yet. The criteria for identifying true positivity within each serum sample was established as the necessity for a positive result from at least two commercially available serological methods. Additionally, the concordance of these methods was examined to address the shortage of available data in the existing literature on leishmaniasis screening. The study also examined potential risk factors based on patient demographics, and the results were deliberated in conjunction with relevant data from the Mediterranean region.

## 2. Materials and Methods

### 2.1. Study Design

As part of our research, we utilized specimens provided by the National AIDS Reference Center of Southern Greece in 2020, obtained from individuals newly diagnosed with HIV and who had not yet received any treatment. The minimum number of necessary samples to meet the desired statistical constraints was estimated at approximately 115. However, to increase the robustness and flexibility of our statistical analysis, we selected a sample size of 155 specimens. Of these, 117 specimens were collected between 2019 and 2020, with the remaining collected in previous years, resulting in a total of 155 specimens. Upon receipt, the specimens maintained at −80 °C for long-term stability were aliquoted to prevent repeated freeze–thaw cycles and then stored at −20 °C. The experimental phase of our study was carried out at the National AIDS Reference Center of Southern Greece, as well as the Laboratory of Molecular Microbiology and Immunology, which is a constituent of the Department of Medical Laboratories at the School of Health Sciences and Welfare of the University of West Attica.

### 2.2. Laboratory Techniques

The serum samples were assessed for IgG antibodies targeting the protozoan *L. infantum* using ELISA, IFAT, and WB assays. All methods were performed according to the manufacturers’ instructions. All reagents and sera to be tested were allowed to come to room temperature before use.

#### 2.2.1. Enzyme-Linked Immunosorbent Assay (ELISA)

The ELISA method was employed to detect IgG antibodies against *L. infantum*, utilizing the *Leishmania infantum* IgG ELISA kit (Demeditec Diagnostics GmbH, Kiel-Germany). The microtiter plates were coated with specific *L. infantum* antigens, and the samples were suitably diluted to 1 + 100. A horseradish peroxidase (HRP) labelled conjugate was utilized, and positive outcomes were ascertained via incubation with TMB substrate. The reaction was stopped by adding 0.2 mol/L sulfuric acid. The absorbance was measured at a test wavelength of 450 nm, while a reference filter was set to 620 nm to rectify any non-specific absorbance that might have occurred. The method’s validity was determined based on the absorbance of the substrate blank, which was required to be less than 0.100, and the negative control, which was required to be less than 0.200, with the cut-off absorption ranging from 0.150 to 1.300. Furthermore, the positive control would be rejected if it exceeded the cut-off value.

#### 2.2.2. Indirect Immunofluorescence Antibody Test (IFAT)

The detection of IgG antibodies against *L. infantum* was carried out using the IFAT method with the *Leishmania* IFA IgG Vircell MICROBIOLOGISTS kit (Granada, Spain). The kit comes with wells coated with *L. infantum* antigens developed in RPMI-1640 medium. The serum samples were diluted to 1:40 and 1:80, and FITC-conjugated IgG was used. A positive outcome was determined by the presence of peripheral, cytoplasmic, and intense fluorescence in promastigotes (Supplementary File S1).

#### 2.2.3. Western Blot (WB)

The LDBIO *LEISHMANIA* WB IgG assay, developed by LDBIO Diagnostics (Lyon, France), was utilized to detect IgG antibodies against *L. infantum*. The methodology involves the separation of *L. infantum* antigens via electrophoresis and their binding by electroblotting to nitrocellulose membranes. An alkaline phosphate–anti-human IgG conjugate is subsequently utilized, and the addition of NBT/BCIP substrate results in the formation of specific bands. The appearance of bands at 14 kDa and/or 16 kDa indicates a positive test outcome.

### 2.3. Data Analysis

In our study, we utilized the qualitative outcomes (positive, negative, or ambiguous/gray zones in some cases) for each of the WB, ELISA, and IFAT immunoassays regarding *L. infantum* IgG detection. Given the qualitative nature of our data, we evaluated the overall method convergence/agreement using Fleiss’ kappa statistical test and the pairwise method agreement using Cohen’s kappa statistical test. In order to assess the statistical significance of the relationships between the given demographic characteristics and *L. infantum* IgG detection in our HIV+ patients, statistical analysis was performed using the Chi-squared (χ^2^) test, with a significance level of 95%, and the Wilson–Brown method to compute confidence intervals (C.I.). Additionally, the relative risk factor was employed to determine the likelihood of *L. infantum* IgG detection among various groups of our HIV+ patients and was calculated by dividing the probability of *L. infantum* IgG detection in the exposed group by the probability in the non-exposed group. The data were organized into 2 × 2 contingency tables for each characteristic (gender, nationality, and intravenous drug use), and each table categorized subjects based on their exposure status (e.g., exposed vs. not exposed to intravenous drug use) and the presence of the event of interest (in this case, *L. infantum* IgG detection).

## 3. Results

### 3.1. Demographics of HIV+ Patients

The 155 patients were an average age of 41 years old. Out of the 155 HIV+ patients examined, 111 (71.6%) were male, 42 (27.1%) were female, and 2 (1.3%) were unclassified (Supplementary File S2). Furthermore, 90 HIV+ patients (58.1%) had Greek nationality, while 62 (40%) had foreign nationality. In terms of HIV testing, 111 (71.6%) were tested for diagnostic reasons, 21 (13.6%) were tested because they were intravenous drug users, and 20 (12.9%) were tested for other preventive reasons.

### 3.2. Laboratory Detection

**Figure 1:**
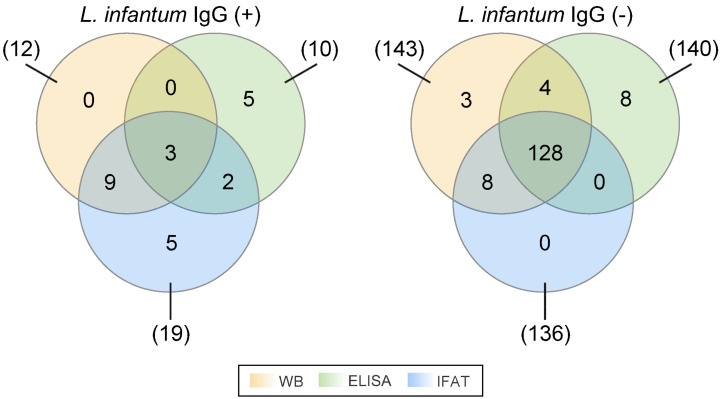
Symmetric three-set Venn diagram of the *Leishmania infantum* immunoglobulin G (IgG) detection results, output by the three examined assays, namely, Western blot (WB), enzyme-linked immunosorbent assay (ELISA), and indirect immunofluorescence antibody test (IFAT). All possible intersections and exclusive regions of each different set of positive [*L. infantum* IgG (+)] or negative [*L. infantum* IgG (−)] assay results have been drawn and colored accordingly. The displayed numbers between the parentheses represent the total number of positive or negative results of the respective assay designated by a line, while the numbers within the colorful regions correspond to the number of positive or negative assay results that are unique to a specific assay or union of assays.

For all of the 155 sampled HIV+ patients, we tested for IgG antibodies against *L. infantum* using three methods: ELISA, IFAT, and WB (Figure 1). The ELISA method yielded 10 (7.5%, C.I. 95% 3.5–11.5%) positive samples, the IFAT method yielded 19 (13.2%, C.I. 95% 8.0–18.4%) positive samples, and the WB method yielded 12 (8.8%, C.I. 95% 4.5–13.0%) positive samples. To ensure the accuracy of the results, a true positive was defined as a sample that tested positive with at least two of the methods, in agreement with the current literature. The findings showed that three samples (3.0%, C.I. 95% 0.7–5.5%) tested positive with all three methods, nine samples (6.9%, C.I. 95% 3.1–10.7%) tested positive with IFAT and WB, and two samples (2.5%, C.I. 95% 0.4–4.6%) tested positive with ELISA and IFAT. Overall, 14 out of 155 samples (10.0%, C.I. 95% 5.5–14.6%) tested positive for IgG antibodies against *L. infantum* with at least two methods. Additionally, five samples (4.4%, C.I. 95% 1.4–7.3%) produced doubtful results (grey zones) with ELISA, which were considered neither negative nor positive in the analysis (Supplementary File S2).

### 3.3. Statistical Analysis

**Figure 2:**
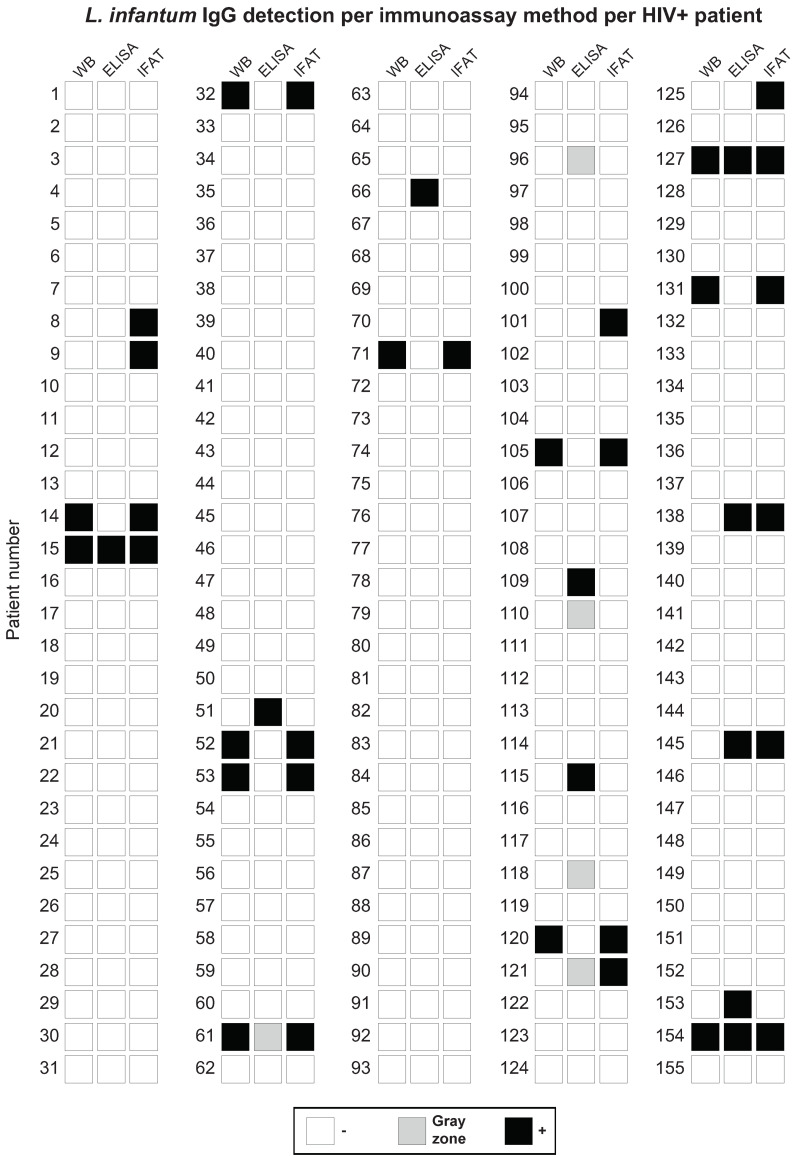
Heatmap figure illustrating the results of *L. infantum* IgG detection for each one of the 155 examined HIV+ patients (− = IgG negative, gray zone = ambiguous result, + = IgG positive) after the implementation of the three studied immunoassay methods, namely, Western blot (WB), enzyme-linked immunosorbent assay (ELISA), and immunofluorescence antibody test (IFAT). The appropriate color legend has been applied (*L. infantum* IgG negative samples in white, ambiguous *L. infantum* IgG detection in light grey, *L. infantum* IgG positive samples in black), highlighting the convergence/agreement of *L. infantum* IgG detection results per immunoassay method per HIV+ patient.

Overall and pairwise agreement between the examined WB, ELISA, and IFAT methods for *L. infantum* IgG detection was determined by Fleiss’ kappa and Cohen’s kappa statistical tests, respectively (Table 1). In total, 131 out of 155 cases (83.7%, C.I. 95% 78.0–89.4%) showed concordance among the three examined methods for *L. infantum* IgG detection, returning moderate overall agreement after statistical analysis, with a Fleiss’ kappa value of 0.42. Similarly, the pairwise method agreement analysis returned (i) 148 out of 155 cases (94.4%, C.I. 95% 91.00–97.8%) concordant between WB and IFAT with a Cohen’s kappa value of 0.75, suggesting substantial agreement; (ii) 135 out of 155 cases (86.2%, C.I. 95% 80.9–91.5%) concordant between WB and ELISA with a Cohen’s kappa value of 0.20, indicating slight agreement; and (iii) 133 out of 155 cases (84.9%, C.I. 95% 79.4–90.4%) concordant between ELISA and IFAT with a Cohen’s kappa value of 0.21, corresponding to fair agreement. Visualization of the convergence/agreement of *L. infantum* IgG detection per immunoassay (WB, ELISA, and IFAT) per examined subject was carried out by a heatmap figure (Figure 2).

In addition, statistical analysis of the included demographic and immunological data was performed and revealed a statistically significant relationship between *L. infantum* IgG detection and intravenous drug use of the examined HIV+ patients (Table 2). More specifically, HIV+ patients with former intravenous drug use are more prone to positive *L. infantum* IgG testing than unexposed HIV+ patients to intravenous drug use (3.55 times higher risk, *p* = 0.025). Apart from this, no other statistically significant relationships were reported between the provided demographic and immunological data.

## 4. Discussion

Various immunological tests are available for diagnosing VL, as it elicits a significant humoral response [15]. In South America, serological tests are considered the primary diagnostic tool for VL [31], and all leishmaniasis reference diagnostic laboratories in North America include serology in their testing for leishmaniasis [23].

The accuracy of serological diagnostic techniques is subject to variation, dependent upon a range of factors, including the method employed; the type, source, and purity of the antigen; the cut-off values for determining a positive result; the location of the study concerning the diversity of parasites; and the nutritional and immunological status of patients, which can significantly impact their antibody production capacity [15,23,33]. There is no agreed-upon minimum requirement for sensitivity and specificity rates for diagnosing VL [31]. The antigens used in diagnostic tests can be whole or soluble promastigotes, organ smears containing amastigotes, or recombinant antigens.

Antigenic diversity among parasites can trigger various immune responses in the host that may not be targeted by a particular diagnostic method [37]. Comparing and interpreting results from different studies can be difficult due to variations in the techniques and the use of different strains and antigens [33]. In cases of co-infection, the humoral response may be attenuated, resulting in low antibody production and false-negative test results [15,20]. Studies indicate that antibody levels against leishmaniasis in HIV+ patients are significantly lower, up to 50-fold, and about 40% of the affected individuals may not have detectable antibodies [10,17]. It should be noted that this percentage pertains solely to patients who exhibit clinical signs of leishmaniasis [20].

During the Fifth Consultative Meeting on *Leishmania*–HIV co-infection, an algorithm was proposed for diagnosing VL in HIV+ patients. The algorithm suggests that in cases where there is a clinical suspicion of VL, samples of blood, skin, or lymph nodes should be taken. Immunochromatographic rapid tests can be conducted, but these tests have limitations in endemic regions due to false positives and low sensitivities in HIV+ patients [15,31]. Alternatively, non-invasive techniques for microscopy can be used, followed by at least two serological tests if the results are negative. Treatment for leishmaniasis should be initiated if the serological tests are positive. If the results are negative, invasive samples (such as bone marrow or spleen) can be tested using PCR, microscopy, or culture [17].

The Infectious Diseases Society of America (IDSA) and the American Society of Tropical Medicine and Hygiene (ASTMH) guidelines recommend using multiple diagnostic approaches to increase the chances of positive *Leishmania* results, and serologic testing is suggested for individuals with suspected VL who cannot undergo or have negative results in definitive diagnostic tests for the parasite [23]. Additionally, the Brazilian Ministry of Health defines a case of VL as the presence of clinical signs with confirmation of diagnosis through serologic and/or parasitological tests [36].

Therefore, the presence of serum antibodies against *Leishmania* spp. alone cannot be considered synonymous with VL disease. However, a positive serological test has diagnostic value when combined with the clinical case definition [33].

In HIV+ patients, the parasites can persist even years after receiving treatment for leishmaniasis. As a result, relapses are pretty standard. Continual use of invasive techniques for monitoring treatment effectiveness, disease progression, and relapses can pose certain risks to patients [17]. Parasites can be activated as a consequence of immunosuppression caused by subsequent HIV infection [6,10]. Asymptomatic and subclinical infections are the most frequent forms of infection in immunocompetent patients in endemic regions [36]. However, there is currently no gold standard for estimating the prevalence of VL infection in asymptomatic populations. Therefore, different methods should be combined to improve the sensitivity of asymptomatic infection diagnosis [35]. In the case of examining the presence of antibodies in a large part of an asymptomatic population, non-invasive and cost-effective techniques are preferred.

Our study was conducted on patients who were recently diagnosed with HIV and had not yet received HAART or previous treatment for leishmaniasis. Due to the absence of any specific symptoms of the disease, there were no clinical suspicions raised regarding the presence of leishmaniasis before HIV diagnosis. This suggests that these patients were infected with *Leishmania* before acquiring HIV and subsequent immunosuppression. Hence, the absence of symptoms can be explained by their immunocompetent status at the time of *Leishmania* infection. However, as Greece is considered an endemic region for VL, these patients were at risk of leishmaniasis relapse at the time of HIV diagnosis. Therefore, we recommend that leishmaniasis screening be conducted immediately after HIV diagnosis to enable prophylactic treatment for leishmaniasis in addition to antiretroviral therapy.

Comparative evaluation studies are crucial to determining the best diagnostic method for VL, as a false-positive result can result in unnecessary toxic treatment, and a false-negative test result can leave patients with a lethal disease untreated [31]. According to the literature, none of the available diagnostic techniques alone are sufficient to identify all VL cases. The varying diagnostic performance of these tests in different VL regions may be attributed to the origin of the test antigen. Strains of *L. donovani* and *L. infantum* found in different regions are genetically heterogeneous and different [20,37]. Therefore, it is not adequate to classify a method as non-performing if it fails in one area. The location where the method was employed, the parasites circulating in that area, and the antigen utilized by the method must all be considered. Hence, every endemic region should provide tests based on *Leishmania* antigens that correspond to the circulating parasites present in that specific region. Tests based on the rK39 are reliable and are officially employed for the national control strategy in countries such as India [37].

A study evaluated the diagnostic accuracy of serological tests in detecting VL in patients with suspected VL. The study evaluated ELISA and IFAT tests. One of the IFAT tests that was evaluated in this study was the same kit that was used in our study. The results showed that this kit had a sensitivity of 60.5% and a specificity of 92.3% [31]. In another study, ELISA, IFAT, and PCR tests were used to detect asymptomatic *Leishmania* spp. infection in HIV+ patients. A significant proportion of patients were infected with *Leishmania* spp. without developing the disease, and most of them (69.3%) were receiving HAART. A significant difference in the inter-assay agreement was achieved based on IFAT detecting antibodies against surface antigens and the crude *L. infantum* ELISA detecting a wider variety of antibodies directed against soluble antigens [36]. Regarding this research, the immunoassay test results and assessment of agreement among the WB, ELISA, and IFAT methods employed for detecting *L. infantum* IgG revealed intriguing findings (Figure 1, Table 1). Among the total of 155 cases, 131 (84.5%) demonstrated concordance across all three methods (Fleiss’ kappa = 0.42), implying moderate overall agreement. Of note, pairwise method agreement analysis indicated substantial agreement between WB and IFAT methods (Cohen’s kappa = 0.75), distinguishing them from the rest of the pairwise method comparisons, which seemed to be fairly discordant. In our study, ELISA uses specific antigens without prior information, while IFAT employs promastigote forms of *L. infantum*. WB utilizes the 14 kDa and 16 kDa antigens of *L. infantum*.

Leishmaniasis is a disease that is prevalent in Greece and is primarily caused by two types of *Leishmania* parasites—*L. major* and *L. infantum*. Annually, instances of leishmaniasis are reported across the Greek territory, with a significant proportion (50%) in the Attica region [19]. The climatic conditions in Greece are conducive to the proliferation of vector sand flies. These sand flies prefer previously rural areas that have now become urbanized [38]. Greece has a high prevalence of canine leishmaniasis [4], and cases have also been reported in feline populations [39]. However, it is widely believed that the presence of an infected pet does not increase the risk of infection for humans. Instead, the high prevalence of the parasite in stray dog populations poses a higher risk. Additionally, cases of leishmaniasis have been reported in tourists who have recently visited Greece [39], indicating that it is a growing threat to tourists.

As per the data obtained from the directorate of epidemiological surveillance and intervention for infectious diseases of the Hellenic Centre for Disease Control and Prevention, between 2004 and 2018, Greece documented 881 cases of VL, out of which 862 were domestic and 19 were imported. Among the domestic cases, 90% were Greek nationals and 65% were male, with a median age of 42 years for males and 32 years for females. Most imported cases were of Albanian origin, with a median age of 24. The average incidence of VL from 2004 to 2018 was estimated to be 0.5 per 100,000 inhabitants [16].

The incidence of VL–HIV co-infection is believed to be rare, as only a limited number of cases have been reported in the literature [4,5]. In our study, we conducted a serological screening of 155 HIV+ patients, with an average age of 41 years old, mostly men (111 out of 155), and Greek (90 out of 155) in nationality, and 14 positive samples detect4ed for IgG antibodies against *L. Infantum* with at least two immunoassay methods. We also managed to uncover intravenous drug use as a risk factor, with HIV+ intravenous drug users being 3.55 times more likely to return positive *L. infantum* IgG tests compared to their counterparts (Table 2).

In a study conducted in France, amastigote forms were detected in 47 cases of *Leishmania*–HIV co-infection. The study revealed that antibodies were detected in 55% of the total patient pool (26 out of 47) through IFAT and ELISA techniques. Moreover, the positivity rate rose to 95% when WB was employed as a confirmatory method. Among the 47 patients, a significant majority of 31 individuals were found to be intravenous drug users [34]. In the 2010 Mandritis outbreak, HIV+ patients accounted for 10% of the total reported cases of VL. It has been observed in xenodiagnosis studies that HIV+ patients can efficiently transmit the protozoan *L. infantum* to the *Phlebotomus perniciosus* vector. This indicates that such individuals have the potential to serve as human reservoirs for the *L. infantum* protozoan. Asymptomatic patients in particular could be “super spreaders” of the parasite, as their skin and venous blood serve as sources of parasites for the sand fly vectors [8,12]. There is also compelling evidence of an alternative artificial cycle wherein the transmission of the parasite can occur through the sharing of contaminated syringes amongst intravenous drug users [10,17,20,40]. Our study is limited in its ability to ascertain the precise mode of *L. infantum* transmission. However, based on the existing literature, we posit that anthroponotic transmission remains a plausible hypothesis.

## 5. Conclusions

The co-infection of VL and HIV presents a significant challenge for VL control and poses a severe threat to public health. There is an urgent need for clinical vigilance and surveillance as leishmaniasis spreads to northern regions and outbreaks re-emerge in endemic areas. In our study, we observed a 9.0% positivity rate among 155 newly diagnosed HIV+ patients, a rate notably exceeding previously documented cases in Greece. Furthermore, our analysis identified intravenous drug use as a substantial risk factor, supporting the plausibility of anthroponotic transmission, which aligns with patterns reported in other endemic regions within the Mediterranean. Serological tests are simple, non-invasive, and cost-effective techniques, preferred for diagnosing asymptomatic and subclinical infections in endemic regions. However, their accuracy is subject to variation, dependent upon a range of factors, including the method employed; the type, source, and purity of the antigen; the cut-off values for determining a positive result; and the nutritional and immunological status of patients. Therefore, to maximize sensitivity, performing at least two different serological methods for each patient is advisable. In our pairwise agreement analysis, WB and IFAT emerged as the methods of choice, demonstrating substantial concordance. In endemic regions, leishmaniasis screening should be conducted immediately after an HIV diagnosis to facilitate the provision of prophylactic treatment for leishmaniasis alongside antiretroviral therapy. Such an approach can help prevent high parasite levels, relapses, and reinfections, and mitigate the symbiotic relationship between the two pathogens, ultimately leading to improved clinical outcomes for these patients with high mortality rates.

## Figures and Tables

**Table 1:** Summary of the overall and pairwise agreement analysis between the examined Western blot (WB), enzyme-linked immunosorbent assay (ELISA), and indirect immunofluorescence antibody test (IFAT) methods for *Leishmania infantum* immunoglobulin G (IgG) detection, accompanied by their 95% confidence intervals in parentheses. Cohen’s kappa and Fleiss’ kappa statistical tests were performed, respectively, and their reported values represent kappa < 0: no agreement, 0 < kappa < 0.20: slight agreement, 0.21 < kappa < 0.40: fair agreement, 0.41 < kappa < 0.60: moderate agreement, 0.61 < kappa < 0.80: substantial agreement, and 0.81 < kappa < 1.00: almost perfect agreement.

Pairwise Agreement						Overall Agreement	
WB & IFAT Cases (%)	WB & ELISA Cases (%)	ELISA & IFAT Cases (%)	WB & IFAT Cohen’s Kappa	WB & ELISA Cohen’s Kappa	ELISA & IFAT Cohen’s Kappa	WB & ELISA & IFAT Cases (%)	Fleiss’ Kappa
148 (94.4%, C.I. 95% 91.00–97.8%)	135 (86.2%, C.I. 95% 80.9–91.5%)	133 (84.9%, C.I. 95% 79.4–90.4%)	0.75	0.20	0.29	131 (83.7%, C.I. 95% 78.00–89.4%)	0.42

**Table 2:** Statistical analysis summary after χ^2^ test of the demographic and immunological data sourced from the HIV+ patients of this study. The number of HIV+ patients with the given characteristics is reported accordingly, alongside the calculated relative risk and statistical significance followed by the respective *p*-values in parentheses (noted with “-” for *p* > 0.05 and “*” for *p* < 0.05).

		HIV+ Patients Positive for *L. infantum* IgG (*n*=)	HIV+ Patients Negative for *L. infantum* IgG (*n*=)	Relative Risk	Statistical significance (*p*)
**Gender**	***Female***	4	38	1.32	- (0.7371)
	***Male***	8	103		
**Nationality**	***Non-Greek***	4	58	0.65	- (0.5612)
	***Greek***	9	81		
**Intravenous drug use**	***True***	5	16	3.55	* (0.0250)
	***False***	9	125		

## Data Availability

The original immunological data presented in the study are included in the Supplementary Materials. Patient demographics are unavailable due to ethical restrictions.

## Supplementary Materials

The following supporting information can be downloaded at: https://www.mdpi.com/article/10.3390/microorganisms12071397/s1, Supplementary File S1: Confocal microscopy images; Supplementary File S2: Immunological data.
