# Supplementary material for: Serological Screening and Risk Factors Associated with Leishmania infantum Positivity in Newly Diagnosed HIV Patients in Greece
Source: Microorganisms. 2024 Jul 10;12(7):1397. doi: 10.3390/microorganisms12071397 (PMC11279118; doi:10.3390/microorganisms12071397)
Supplement: Supplementary file 1 [file microorganisms-12-01397-s001.zip › Supplementary_file_1.pptx]

## Slide 1
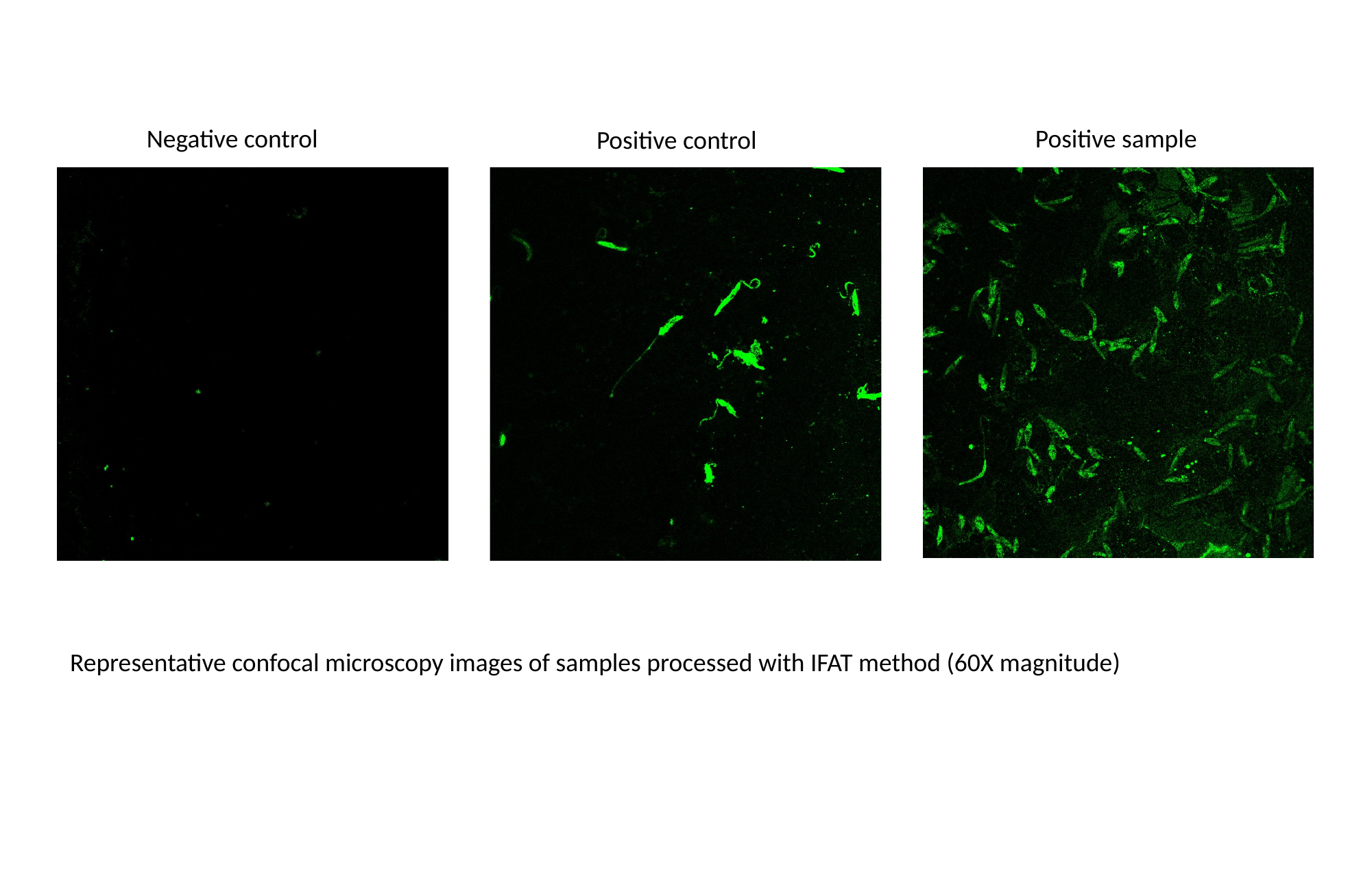

Negative control
Positive sample
Positive control
Representative confocal microscopy images of samples processed with IFAT method (60X magnitude)
